# Prevalence and factors associated with hypertension among children attending pre-schools in Dar es Salaam, Tanzania

**DOI:** 10.1186/s13052-025-01841-y

**Published:** 2025-03-19

**Authors:** Jida Said, Nahya Salim, Peter P. Kunambi, Francis Furia

**Affiliations:** 1 Department of Pediatrics and Child Health, School of Clinical Medicine, Muhimbili University of Health and Allied Sciences, Dar Es Salaam, Tanzania; 2 St. Joseph’s Mission Hospital, Peramiho, Ruvuma Tanzania; 3 Department of Clinical Pharmacology, School of Biomedical Sciences, Muhimbili University of Health and Allied Sciences, Dar Es Salaam, Tanzania

**Keywords:** Prevalence, High blood pressure, Pre-school children, Tanzania, Early childhood, Low birth weight

## Abstract

**Background:**

Childhood hypertension has become a public health problem due to its increasing prevalence and complications; the high prevalence is noted to mirror an increase in obesity among children. Hypertension in children is frequently undiagnosed due to challenges in getting appropriate cuff sizes and interpretation of the readings. Several studies have been carried out among children however; most of available information is focused on hypertension among older children and adolescents.

**Methods:**

A cross sectional study was conducted in 2 districts of Dar es Salaam region namely Ilala and Kinondoni from October to November 2020. Children aged 2–5 years attending pre-schools in these districts meeting the inclusion criteria and whose parent/guardian signed informed consent were included. Questionnaires were used to collect socio-demographic characteristics; anthropometric and three oscillometric single- occasion blood pressure measurements were taken. The average blood pressure was compared to the standard charts for age and sex provided by the American Academy of Pediatrics 2017 to determine the blood pressure category of the child.

**Results:**

A total of 1083 children fulfilled the eligibility criteria and were enrolled into the study, 51.3% (556/1083) of participants were males and the median age was 4 years (IQR 3–5). Blood pressures for 252 (23.3%) participants were in the high blood pressure range (19.8% with elevated blood pressure and 3.5% with hypertension). No significant gender difference was observed among those with high blood pressure. Factors that were noted to be significantly associated with elevated blood pressure included low birth weight (*p* = 0.036), increasing age (*p* = 0.032) and body mass index (*p* < 0.001).

**Conclusion:**

High prevalence of elevated blood pressure in this population of pre-school aged children is alarming. Low birth weight, increasing age and body mass index were significantly associated with elevated blood pressure.

## Background

Over the recent decades childhood hypertension has become a widely investigated topic due to its increasing prevalence and complications. The worldwide prevalence of hypertension in children and adolescents is estimated to be between 2 and 4% [1] while in African countries the prevalence established through systematic reviews is estimated to be between 5% and 7.45% [2, 3]. Differences in blood pressure (BP) measurement procedures may lead to variations in prevalence estimates. A study conducted among adolescents in Uganda and Tanzania using a single occasion BP measuring approach reported a prevalence of 11% [4] while another study carried out among adolescents in Tanzania using 24 h ambulatory BP monitoring approach reported a prevalence of 2.6% [5]. Reports of high prevalence rates of hypertension (8.5–10.8%) have been documented from studies involving primary school children aged 6–17 years in Tanzania [6, 7]. There are various reports of wide variation in the prevalence of hypertension among children aged 2–6 years ranging from 1.9% to 19.9% [8, 9]. Secondary hypertension is reported to be more common among preadolescent children and is predominantly attributed to renal diseases [10]. A wide spectrum of complications result from childhood hypertension involving multiple organs including heart, brain, kidneys, eyes and blood vessels. Left ventricular hypertrophy is the most prominent clinical evidence of end-organ damage in childhood hypertension [11].

Data on hypertension among younger children are scarce in the sub-Saharan African region, particularly for children aged below five years. Few studies conducted among pre-school children have documented existence of elevated blood pressure [8, 12, 13], however, factors which are associated with it are variable, inconsistent and not well studied [8, 12, 14]. Lack of data and greater variability in reports from countries in this region justified the need for this study to be conducted in Tanzania. Understanding the magnitude and factors for hypertension in this age group will raise awareness and support planning and implementation of appropriate preventive strategies. This study was therefore conducted to estimate the prevalence of hypertension and its associated factors among children attending pre-schools in Dar es Salaam, Tanzania.

## Methods

### Definitions of concepts

Term baby—Is a baby born between 37 complete weeks and 42 weeks of gestation age [15].

Preterm baby – Is a baby born before 37 complete weeks of gestation age [15].

Low birth weight baby – Is a baby born with weight less than 2.5 kilograms [16].

Normal birth weight – Is a birth weight equal to or greater than 2.5 kilograms not exceeding 4.0 kilograms [16].

High birth weight - Is a birth weight equal to or greater than 4.0 kilograms [16].

Passive smoker—a child involuntarily inhaling cigarette smoke in the household.

Underweight—Body Mass Index less than 5.^th^ age and sex specific percentile [17].

Normal weight – Is a Body Mass Index between 5th and less than 85.^th^ age and sex specific percentile [17].

Overweight – Is a Body Mass Index between 85th and less than 95.^th^ age and sex specific percentile [17].

Obese—Body Mass Index equal to or greater than 95.^th^ age and sex specific percentile [17].

History of hypertension in family- Is the presence of hypertension in the first and second-degree relatives.

### Study design and participants

We conducted a community based cross sectional study among children aged 2–5 years attending preschools in two of the five districts of Dar es Salaam region, namely Ilala and Kinondoni. The two districts cover about 53% of the whole region’s area. Dar es Salaam is the most populated city in Tanzania. Children who were uncooperative during anthropometric measurements were excluded from the study.

### Study sample size, sampling technique and procedures

Kish Leslie formula was used to calculate the sample size based on the following assumptions; expected proportion of 50% due to the inconsistence in findings from previous studies, significance level of 95% and a desired precision of 3% with a non-response rate of 20%.

Ilala and Kinondoni districts were conveniently selected, and a sampling frame with a list of preschools in these two districts with clear categorization into either public or privately owned was prepared. A proportional random sampling technique was used to obtain schools included in this study.

Data was collected using self-administered structured questionnaire, which was developed in English and later translated to Swahili language. Information regarding socio-demographic characteristics of study participants and risk factors associated with hypertension were collected. Initial visit was made and consent forms and questionnaires were included in the children’s homework packages and distributed to parents who filled them at their homes.

All students found in the selected schools meeting the inclusion criteria and had filled and returned questionnaires and consent forms by parents were enrolled in the study. Children who were not cooperative during anthropometric measurements were excluded from the study.

### Weight and height measurement

The principal investigator and 3 research assistants (intern doctors) who were trained on the study protocol conducted anthropometric and blood pressure measurements. Weight was measured to the nearest 0.1 kg and height to the nearest 0.1 cm using a calibrated mechanical weighing with height scales (SERICO RGZ-160^©^ Shanghai, China). Children removed their shoes during the measurement. Body mass index (BMI) was then calculated as weight in kilograms divided by the square of height in meters (kg/m2). BMI category was defined according to centers for disease control and prevention (CDC) 2000 BMI percentile for age and gender [17] charts whereby BMI < 5th percentile was defined as underweight, BMI ≥ 5th to < 85th percentile as normal weight, BMI ≥ 85th to < 95th percentile as overweight and BMI ≥ 95th percentile as obesity.

### Blood pressure measurement

To keep the children calm, measurements were done in their usual environment (classrooms), researchers wore casual clothing without white coats and the process took place with peers around. Three measurements of BP were taken at the same time by different researchers at least 5 to 10 min apart. Blood pressure was measured on right arm using a digital blood pressure measuring machine (Omron Digital HEM-907^©^, Tokyo, Japan) with appropriate cuff size and the child seated comfortably on a chair. The three readings were averaged and the average reading was compared to the BP tables based on age, gender and height percentile by the AAP 2017 guidelines [18] as follows:

- Elevated Blood Pressure – Blood pressure ≥ 90th percentile to < 95th percentile or 120/80 mm Hg to < 95th percentile.
- Stage 1 Hypertension – Blood pressure ≥ 95th percentile to < 95th percentile + 12 mmHg, or 130/80 to 139/89 mmHg (whichever is lower).
- Stage 2 Hypertension – Blood pressure ≥ 95th percentile + 12 mm Hg, or ≥ 140/90 mm Hg (whichever is lower).

### Data analysis

Data analysis was performed using Statistical Package for Social Sciences (SPSS) version 26. Descriptive statistics including frequency for categorical variables and median and interquartile range (IQR) for numerical variables were used to describe social demographic and clinical characteristics of participants. Univariate and multivariate logistic regression models were used to determine odds ratio (OR), 95% confidence interval (CI) and *p* values for the factors associated with hypertension. All variables reported to be associated with hypertension were entered in a multivariate model where adjusted odds ratio with *p* value < 0.05 were considered statistically significant. Analysis followed the standard cut-offs as narrated in the definitions of terms for the purpose of this study.

## Results

**Fig. 1:**
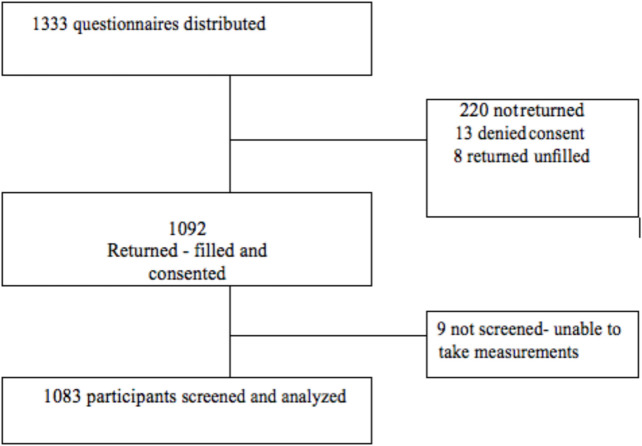
Flow chart of the study participants

A total of 1333 questionnaires and consent forms were distributed to parents, out of these 220 were not returned and 8 were returned but not filled, making response rate of 83%. Thirteen parents did not consent hence 1092 children were enrolled into the study. During data collection, 9 children were excluded from the study because they were not calm enough to allow blood pressure measurement leaving 1083 participants whose data were analyzed. Figure 1 below shows the flow of participants recruited.

### Socio-demographic characteristics of the study participants

Descriptive statistics of the study participants are summarized in Table 1. The median age was 4 years (IQR 3-5). Out of 1083 participants, 48.7% were females and 51.3% were males. The majority (67.9%) of the study participants (*n* = 735) were residing in Kinondoni. The median age of children’s fathers was 35 years (IQR 32- 40) and mothers 30 years (IQR 27- 34). Only 30.7% of the fathers were formally employed.

**Table 1:** Socio-demographic characteristics of the 1083 study participants

Variable	Frequency (n)	Percent (%)
Age of the children (years)		
2	69	6.4
3	212	19.6
4	310	28.6
5	492	45.4
Median age of the children in years (IQR)	4 (3, 5)	
Sex of the child		
Male	556	51.3
Female	527	48.7
Residence		
Ilala	321	29.6
Kinondoni	735	67.9
Ubungo	20	1.8
Temeke	7	0.6
Age of father (years)		
20—35	555	51.2
36—45	432	39.9
> 45	96	8.9
Median age of fathers in years (IQR)	35 (32, 40)	
Age of Mothers (years)		
18—25	160	14.8
26—35	704	65.0
> 35	219	20.2
Median age of Mothers in years (IQR)	30 (27, 34)	
Education level of the fathers		
No formal	4	0.4
Primary	328	30.3
Secondary	609	56.3
College/University	142	13.1
Education level of the mother		
No formal	13	1.2
Primary	388	35.8
Secondary	584	53.9
College/ University	98	9.0
Occupation of the father		
Unemployed	58	5.4
Employed	332	30.7
Self employed	600	55.4
Contract worker	69	6.4
Peasant	24	2.2

### Clinical characteristics of the study participants

The majority of the participants (1036/1083, 95.7%) were born at term and 82.9% (898/1083) had a normal birth weight. The distribution of the participant’s current weight was 622 (57.4%) normal weight, 247 (22.8%) underweight, 144 (13.3%) overweight and 70 (6.5%) obese as shown in Table 2 below.

**Table 2:** Clinical characteristics of the 1083 study participants

Variable		Frequency (n)	Percent (%)
Gestation age			
	Term	1036	95.7
	Preterm	47	4.3
Birth weight			
Normal		898	82.9
Low birth weight		120	11.1
High birth weight		65	6
Maternal hypertension during pregnancy			
Yes		44	4.1
No		1039	95.9
Maternal cigarette smoking			
	Never smoked	1068	98.6
	Stopped ≥ 3 months before pregnancy	13	1.2
	Smoked after the pregnancy	2	0.2
Smoking during pregnancy			
	Third trimester	2	0.2
	No	1081	99.8
Number of cigarettes smoked during pregnancy			
	1	2	100
Passive smoking			
	Yes	149	13.8
	No	934	86.2
Adult cigarette smoker			
	Father	95	63.8
	Relative	52	34.9
	Father and Relative	2	1.3
Duration of smoking for father			
	1 – 5 years	44	45.4
	6 – 10 years	27	27.8
	> 10 years	26	26.8
Duration of smoking for relative			
	1 – 5 years	30	57.7
	6 – 10 years	4	7.7
	> 10 years	18	34.6
Place of smoking			
	Home	71	47.7
	Away	69	46.3
	Both home and away	9	6.0
Home smoking			
	Outdoor	34	39.1
	Indoor and outdoor	53	60.9
History of hypertension in family			
	Yes	145	13.4
	No	938	86.6
BMI of the child			
	Under weight	291	26.9
	Normal weight	703	64.9
	Over weight	42	3.9
	Obese	47	4.3

### Prevalence of hypertension

**Fig. 2:**
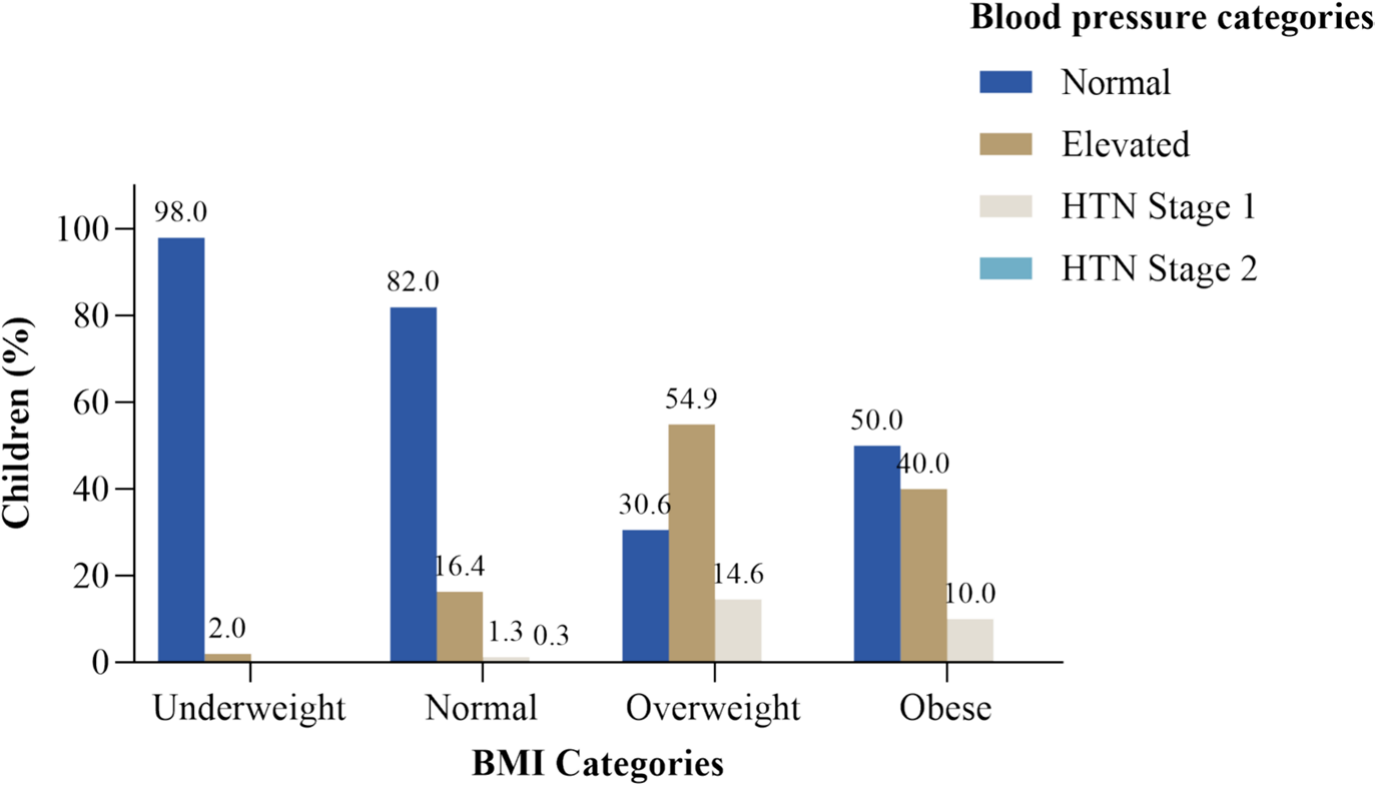
Prevalence of blood pressure categories by BMI categories among children attending preschool, Dar es Salaam –Tanzania, *N* = 1083

Blood pressure in hypertensive range was detected in 38 (3.5%) of the study participants, of which 36 (3.3%) had stage 1 hypertension while 2 (0.2%) had stage 2 hypertension. A total of 215 (19.8%) had blood pressure in elevated blood pressure range at the time of the study. Figure 2 summarizes the prevalence of blood pressure categories by BMI categories among the studied preschool children.

### Factors associated with hypertension

The prevalence of hypertension was markedly higher among participants with overweight (69.4%) and obesity (50%) as compared to those with normal weight (18%) and underweight (2%). Participants who were born with low birth weight had a higher prevalence of hypertension (37.5%) compared to those born with normal weight (21.6%) and high birth weight (20.0%) with statistically significant differences as shown in Table 3 below.

**Table 3:** Socio-demographic factors associated with hypertension

Hypertensive			
**Variable (Risk factor)**	**Yes (%)**	**No (%)**	***p*-value**
Age of the child			
2	16 (23.2)	53 (76.8)	0.297
3	42 (19.8)	170 (80.2)	
4	83 (26.8)	227 (73.2)	
5	111 (22.6)	381 (77.4)	
Sex			
Male	132 (23.7)	424 (76.3)	0.706
Female	120 (22.8)	407 (77.2)	
Residency			
Ilala	82 (25.5)	239 (74.5)	0.280
Kinondoni	164 (22.3)	571 (77.7)	
Ubungo	6 (30.0)	14 (70.0)	
Temeke	0 (0.0)	7 (100)	
Birth weight			
Normal	194 (21.6)	704 (78.4)	**< 0.001**
Low	45 (37.5)	75 (62.5)	
High	13 (20.0)	52 (80.0)	
Gestation age			
Term	242 (23.4)	794 (76.6)	0.741
Preterm	10 (21.3)	37 (78.7)	
Maternal hypertension during pregnancy			
Yes	8 (18.2)	36 (81.8)	0.415
No	244 (23.5)	795 (76.5)	
BMI of the child			
Normal	112 (18.0)	510 (82.0)	**< 0.001**
Obese	35 (50.0)	35 (50.0)	
Overweight	100 (69.4)	44 (30.6)	
Underweight	5 (2.0)	242 (98.0)	
History of Hypertension in family			
Yes	25 (17.2)	120 (82.8)	0.065
No	227 (24.2)	711 (75.80	
Passive smoking			
Yes	35 (23.5)	114 (76.5)	0.945
No	217 (23.2)	717 (76.8)	
Maternal cigarette smoking			
Never smoked	252 (23.6)	816 (76.4)	0.113
Stopped ≥ 3 months before pregnancy	0 (0.0)	13 (100)	
Smoked after pregnancy	0 (0.0)	2 (100)	

### Relationship between hypertension and clinical features

Univariate analysis showed overweight participants were ten times more likely to have blood pressure in hypertensive range compared to those with normal weight (crude odds ratio (cOR) 10.35, 95% CI 6.87 – 15.58, *p* = < 0.001). Participants who were obese were 4.6 times more likely to have blood pressure in hypertensive range compared to those with normal weight (cOR 4.55, 95% CI 2.73 – 7.59, *p* = < 0.001). Participants who were underweight were less likely to have blood in hypertensive range compared to those with normal weight (cOR 0.09, 95% CI 0.04- 0.23, *p* = < 0.001). No statistically significant association was noted with hypertension for other factors.

At multivariate analysis level, an increase in age was found to be independently associated with hypertension, and participants aged 4 years and 5 years were 2.3 times and 2.2 times more likely to have blood pressure in hypertensive range as compared to those aged 2 years, respectively (adjusted odds ratio) aOR 2.19, 95% CI 1.08 – 4.44, *p* = 0.030).

Overweight and obese participants were 7.7 and 3.3 times more likely to have blood pressure in hypertensive range than those with normal weight, respectively (aOR 11.77, 95% CI 7.66 – 18.08, *p* = < 0.001 and aOR 5.65, 95% CI 3.28- 9.73, *p* = < 0.001). Those who were underweight were ten times less likely to have blood pressure in hypertensive range compared to those having normal weight (aOR 0.09, 95% CI 0.04 – 0.22, *p* = < 0.001). Table 4 summarizes the univariate and multivariate analysis results of factors associated with hypertension in the studied children.

**Table 4:** Univariate and multivariate analysis of factors associated with hypertension

	Univariate analysis			Multivariate analysis		
**Variable**	**cOR**	**95% CI**	***p* -value**	**aOR**	**95% CI**	***p* – value**
Age (years)						
5	0.97	0.53 – 1.75	0.907	2.15	1.05 – 4.38	**0.036**
4	1.21	0.66 – 2.24	0.540	2.34	1.13 – 4.84	**0.022**
3	0.82	0.43 – 1.57	0.548	1.29	0.60 – 2.77	0.510
2	Ref					
Sex						
Female	0.95	0.71 – 1.26	0.706	1.00	0.71 – 1.40	0.997
Male	Ref					
Birth weight						
Low weight	2.18	1.46 – 3.26	**< 0.001**	1.84	1.05 – 3.21	**0.032**
High weight	0.91	0.48 – 1.70	0.761	0.81	0.37 – 1.78	0.603
Normal weight	Ref					
Gestation age						
Preterm	0.89	0.44 – 1.81	0.741	0.47	0.18 – 1.22	0.120
Term	Ref					
Maternal hypertension during pregnancy						
Yes	0.72	0.33 – 1.58	0.417	0.83	0.31 – 2.22	0.709
No	Ref					
BMI of the child						
Obese	4.55	2.73 – 7.59	**< 0.001**	5.23	3.03 – 9.04	**< 0.001**
Overweight	10.35	6.87 – 15.58	**< 0.001**	11.26	7.29 – 17.38	**< 0.001**
Underweight	0.09	0.04 – 0.23	**< 0.001**	0.09	0.03 – 0.23	**< 0.001**
Normal weight	Ref					
History of HTN in family						
Yes	0.65	0.41 – 1.03	0.067	0.57	0.33 – 0.98	**0.043**
No	Ref					
Passive smoking						
Yes	1.01	0.68 – 1.53	0.948	0.92	0.57 – 1.50	0.749
No	Ref					

*Key cOR *crude odds ratio, *aOR *adjusted odds ratio, Ref: Reference group

## Discussion

The current study presents the findings of blood pressure profile among preschool children from an urban setting Dar es Salaam, Tanzania. Hypertension prevalence among these children was alarming and factors that were associated with increased risk were low birth weight, increasing age and overweight/obesity. The prevalence of elevated blood pressure and hypertension was found to be 19.8% and 3.5%, respectively.

We observed the prevalence of hypertension at 3.5%, which is comparable to another study conducted in similar setting [19]. However the prevalence found in our study is lower than previously reported prevalence in high income [12, 20]and upper-middle income countries [9]which could be explained by the differences in geographical location, living conditions and ethnicity of the studied populations. The prevalence found in our study was higher than that found in a study of similar age population [8], the difference in findings could be due to variations in criteria used in defining hypertension and methodological differences in blood pressure (BP) measurement, such as the type of device used and the number of measurements taken.

In Tanzania, no specific study was found documenting prevalence of hypertension among preschoolers. Studies done in primary school children aged 6–17 years report a higher prevalence of hypertension, between 8.5% and 10.8% [9,10)]. However, the findings are incomparable due to differences in age of the studied populations and definition of hypertension used.

On multivariate analysis, an increase in age was associated with a risk of hypertension with higher prevalence among the 4- and 5-year-olds. This finding is comparable to another study done in similar age group [3]. This finding can partly be attributable to increase in weight with age. In contrast to this, Crispim et al [9] found a higher prevalence in the youngest age group (2 years) which could be due to greater anxiety on the occasion of measuring BP compared to the older age groups [21].

No significant association was found between sex and elevated blood pressure in this study, a finding similar to others studies done in similar population in both rural and urban areas [11,17,19)]. However findings related to the association between sex and BP in preschoolers are inconsistent, with Simonetti and colleagues [22] reporting lower systolic blood pressure (SBP) in females than males while Rice and colleagues [20] reported higher SBP in females. The difference in findings could be attributed to the variation in blood pressure measurement techniques used.

Both overweight and obesity were found to be independently and significantly associated with elevated blood pressure, a finding similar to other international studies [9, 12, 19, 22–25]. Elevations in blood pressure with weight gain in children could be attributed by the increase in heart rate and cardiac output, which activates the sympathetic nervous system and influences insulin resistance. The Bogalusa Heart Study has evidenced the association of overweight and high BP, which can lead to end organ damage, predisposing to cardiovascular disease development in adulthood. Therefore, actions preventing weight gain are important to avoid systemic arterial hypertension appearance in children [26].

Our study did not find any association between gestational age at delivery and elevated blood pressure in contrast to other studies, which showed a higher prevalence in children who were born prematurely at different ages as early as 2.5 years [13, 27–31]. A possible explanation for these contrasting results is the fact that processes associated with intrauterine growth restriction initiate elevated blood pressure, however become amplified later in life as described by Barker et al and Kistner et al. [31, 32].

A statistically significant association was found between low birth weight and elevated blood pressure, a finding similar to other studies [22, 26]. One of the suggested mechanisms that links birth weight and blood pressure levels and fluctuations through life course is the increased sympathetic nervous system activity established in utero [26].

Children with no family history of hypertension were found to have a higher prevalence of elevated blood pressure (21.7%) compared to those with a family history of hypertension (12.4%). In contrast, previously conducted studies [22, 33] showed a higher prevalence of childhood hypertension in children with a family history of hypertension. The discrepancy in these findings could be due to the fact that children with a positive family history of hypertension and age > 6 years usually have primary hypertension [34, 35] while our study included 2–5 years old children in whom secondary hypertension is the leading cause [36, 37].

Children born from mothers with no history of hypertension during pregnancy were found to have a higher prevalence of elevated blood pressure (21.7%) compared to those with mothers with history of hypertension during pregnancy (12.4%). In contrast, other studies [38–41] showed hypertensive disorders in pregnancy are associated with higher blood pressure in the offspring, whereby early pregnancy appeared to be the period with the most influence on childhood blood pressure [39, 42]. This discrepancy in findings could be due to the fact that most of the previous studies outcomes were obtained when children were somewhat older than in our study, starting from 6 years [39] to adolescence [43], and associations may emerge with further follow up. Furthermore, the number of mothers with history of hypertension during pregnancy was relatively small and this might have led to lack of power for the association of hypertension during pregnancy and childhood blood pressure. Recall bias can also not be excluded.

We found no association between passive cigarette smoking and elevated blood pressure, a finding similar to Crispim et al [9]. Other studies showed a higher prevalence of elevated blood pressure in children exposed to passive cigarette smoking compared to those who were not [22, 44]. Maternal cigarette smoking during pregnancy had no association with elevated blood pressure a finding similar to Crispim et al [9]. Previous studies observed a higher prevalence of elevated blood pressure in children with a history of maternal smoking during pregnancy [45–48]. The difference in findings could be due to geographical location, living conditions, and cultural practices, biased self-reporting of smoking during pregnancy and ethnicity of the studied populations.

Racial disparity in hypertension exists in both children and adolescents whereby the prevalence of hypertension is greater in blacks than whites [10, 49, 50]. Parameters proposed to contribute to this disparity are: presence of higher salt sensitivity and higher rates of overweight and obesity in blacks which contributes to higher blood pressures than in whites [51]. Geographical location and living conditions also affect blood pressure whereby urban dwellers have higher prevalence of hypertension than non-urban dwellers [52] while children with insufficient levels of moderate to vigorous physical activity are at significantly greater risk for elevated SBP than sufficiently active counterparts [12].

### Strength of the study

This is the first study in our setting which provides the magnitude of hypertension in preschool children in urban settings of Dar es Salaam; a highly populated region in Tanzania with a larger sample size.

### Limitations of the study

The use of oscillometric BP measuring device instead of the auscultation, which is required for diagnosis confirmation, could have caused overestimation of systolic and diastolic BP as compared with values obtained by auscultation [53]. Three blood pressure measurements were taken on one occasion 5–10 minutes apart, this method may lead to elevated blood pressure in this population being overestimated due to anxiety. Participants who were found to be hypertensive were not screened for possible secondary causes. There is a possibility of recall bias in some questions asked in the questionnaires, together with the probability of socially acceptable responses for example regarding maternal cigarette smoking during pregnancy due to cultural practices, which may have affected our findings.

## Conclusion

The prevalence of hypertension among preschool children aged 2 – 5 years in Dar es Salaam, Tanzania is 3.5% and that of elevated blood pressure (previously known as pre-hypertension) is 19.8%. There is a significant association between hypertension and low birth weight, increasing age, overweight and obesity. We recommend regular screening for hypertension among preschool children especially those born with low birth weight, and those who have overweight/obesity throughout childhood to detect and manage hypertension early. Further studies with BP measurements on different occasions or ambulatory BP monitoring approach should be conducted to better estimate the magnitude of hypertension, and determine other factors not investigated in this study.

## Data Availability

The datasets used and/or analyzed during the current study are available from the corresponding author on reasonable request.

## Abbreviations

AAP: American academy of pediatrics
BMI: Body mass index
BP: Blood pressure
DBP: Diastolic blood pressure
SBP: Systolic blood pressure
